# Exploring Factors Related to Self-Advocacy Among Patients Receiving Maintenance Hemodialysis in China: A Qualitative Study Based on the COM-B Model

**DOI:** 10.3390/healthcare14213758

**Published:** 2026-11-04

**Authors:** Jiayu Wei, Jing Yu, Huisi Tang, Yiting Zhuo, Jialian Huang

**Affiliations:** School of Nursing, Guangdong Pharmaceutical University, Guangzhou 510310, China; 2112419025@stu.gdpu.edu.cn (J.W.);

**Keywords:** maintenance hemodialysis, self-advocacy, COM-B model, influencing factors, qualitative study

## Abstract

Background: Patient-centered care requires patients receiving maintenance hemodialysis (MHD) to communicate their health needs and actively participate in disease management. Self-advocacy is a behavioral process through which patients seek health-related information, express individualized needs, and participate in disease management. Enhancing self-advocacy is essential for advancing patient-centered care in MHD. Objectives: This study sought to investigate the factors influencing self-advocacy among patients receiving MHD. Methods: From February to May 2026, we recruited 18 patients receiving MHD from the blood purification center of a tertiary hospital in Guangzhou, China, using purposive sampling. Guided by the Capability, Opportunity, Motivation–Behavior (COM-B) model, we conducted a descriptive qualitative study. We collected data through semi-structured interviews and analyzed them using inductive–deductive thematic analysis. Results: Guided by the COM-B model, the findings encompassed three dimensions: capability, opportunity, and motivation. Regarding capability, patients experienced cognitive overload and difficulty translating experiences into expression, while some acquired proactive coping strategies. Regarding opportunity, family relationships constrained patient voice, whereas ongoing patient–provider relationships fostered empowerment. Regarding motivation, patients showed sensitivity to relational costs and awareness of financial burden, while also demonstrating a sense of responsibility for their health. Conclusions: The findings revealed that multiple factors across the dimensions of capability, opportunity, and motivation influenced self-advocacy among patients receiving MHD. Promoting self-advocacy requires not only improving patients’ ability to understand health information and communicate effectively, but also strengthening family support, improving patient–provider interactions, and supporting patients’ sense of responsibility for their health. Future research could explore targeted support strategies based on these findings and evaluate their feasibility and effectiveness in dialysis care.

## 1. Introduction

End-stage kidney disease (ESKD) is the last stage of chronic kidney disease, in which there is irreversible loss of kidney function and the need for kidney replacement therapy, such as dialysis or transplantation, to sustain life [1]. According to the Chinese National Renal Data System, the annual incidence of treated ESKD in China increased from 63.5 per million population in 2012 to 134.9 per million in 2022 [2].Owing to the limited availability of kidney donors, high medical costs, and limitations in medical technology, approximately 80% of patients receiving kidney replacement therapy currently undergo maintenance hemodialysis (MHD) [3]. During this period, the total number of patients receiving hemodialysis in China increased from 248,016 to 844,265 [2]. Although MHD can effectively prolong survival, it replaces only part of normal kidney function. Patients therefore experience multiple and cumulative symptom burdens, including pruritus, nausea, and fatigue, which can substantially impair quality of life [4]. Dialysis-related symptom burden is highly subjective and varies considerably between individuals, with symptoms often changing over time [5]. Effective identification and management of these symptoms therefore require patients’ active expression and participation. In addition, patients need to maintain ongoing communication with healthcare teams regarding vascular access management and dialysis prescription adjustments to ensure that treatment plans better align with their health conditions and daily needs [6]. Thus, these persistent treatment burdens and healthcare interactions not only challenge patients’ physical and psychological well-being but also place greater demands on their active participation in disease management and healthcare decision-making.

Self-advocacy refers to the behavioral process through which patients actively seek health-related information, express their needs, and participate in decision-making during disease management [7]. Research has shown that patients with higher levels of self-advocacy have greater awareness of their symptoms, are better able to obtain external support, and are more likely to choose treatment and care approaches that suit their needs, thereby improving their quality of life [8]. As patient-centered care continues to develop, attention has increased to patients’ individual needs and preferences. As an important form of active patient participation in disease management, self-advocacy provides a foundation for implementing patient-centered care [9]. However, self-advocacy among patients receiving MHD in China remains suboptimal [10]. Identifying the factors that shape self-advocacy is therefore essential to develop targeted interventions and promote patients’ active participation in disease management.

Current approaches to promoting patient participation in their care primarily include pre-dialysis and intradialytic health education, self-management support, and shared decision-making [11,12]. However, existing studies have focused mainly on patients’ knowledge, self-management capabilities, or participation in specific treatment decisions, with relatively little attention to how patients actively express their needs and participate in care throughout ongoing dialysis. Self-advocacy is a complex behavioral process shaped by individual factors and external contexts, including patient–healthcare professional interactions and social support. Quantitative approaches may identify associated factors but provide limited insight into how these factors influence the development of self-advocacy [10]. Qualitative research is therefore appropriate for exploring patients’ subjective experiences and the meanings they attribute to self-advocacy. To further organize these influences and explain how they contribute to behavior formation, a theoretical framework is needed [13]. The Capability, Opportunity, Motivation–Behavior (COM-B) model, proposed by Michie et al. [14], conceptualizes behavior as the result of the interaction between capability, opportunity, and motivation. The model has been broadly employed in research of self-management and health promoting behaviors of patients suffering from chronic diseases [15,16], and offers a systematic frame to review factors related to self-advocacy among patients undergoing MHD.

Therefore, this study adopted the COM-B model as its theoretical framework and used a descriptive qualitative design to systematically explore the factors associated with self-advocacy among patients receiving MHD across the three dimensions of capability, opportunity, and motivation. The findings may provide a theoretical basis for developing culturally appropriate interventions.

## 2. Materials and Methods

### 2.1. Study Design

Guided by the COM-B model, this study employed a descriptive qualitative design and used semi-structured interviews to explore the factors influencing self-advocacy among patients receiving MHD. The study was reported in accordance with the Consolidated Criteria for Reporting Qualitative Research (COREQ) checklist [17] (see File S1).

### 2.2. Participants

Purposive sampling was used to recruit patients receiving MHD from the blood purification center of a tertiary Grade A hospital in Guangzhou, China, between February and May 2026. With the assistance of the director and head nurse of the center, the research team introduced the study in the department. The interviewer subsequently approached eligible patients face-to-face in the department and invited them to participate. A total of 18 patients were invited, all invited patients consented, and no participants withdrew from the study. The inclusion criteria were as follows: (1) meeting the diagnostic criteria for end-stage kidney disease [1]; (2) age ≥ 18 years; (3) receiving hemodialysis for ≥3 months [1]; and (4) being conscious and having normal verbal communication and comprehension abilities. Exclusion criterion: Patients participating in clinical intervention trials beyond routine pharmacological treatment and nursing care, including drug trials, medical device trials, and non-pharmacological intervention trials. The sample size was determined based on data saturation [18]. After the 16th interview, the research team conducted an initial analysis and discussion and provisionally considered the data saturated. Following the 17th and 18th interviews, no new data emerged, confirming data saturation.

### 2.3. Theoretical Framework

The COM-B model guided both the development of the interview guide and the data analysis process in this study [14]. The model proposes that behavior arises from the interaction of three components: Capability, Opportunity, and Motivation. Capability refers to an individual’s psychological and physical ability to perform a specific behavior. Psychological capability includes the cognitive abilities required to acquire, understand, and apply information, make judgments, and make decisions. In contrast, physical capability encompasses the physical skills and physiological conditions required to perform the behavior. Opportunity refers to external factors in an individual’s environment that enable or constrain behavior, including physical opportunity (e.g., access to resources, time, and services) and social opportunity (e.g., social norms, interpersonal relationships, and support from others). Motivation refers to the internal processes that drive behavior, including reflective motivation (e.g., beliefs about the value and consequences of behavior, goals, and plans) and automatic motivation (e.g., emotional responses, habits, and impulses). These three components interact to influence behavior initiation and maintenance.

**Figure 1:**
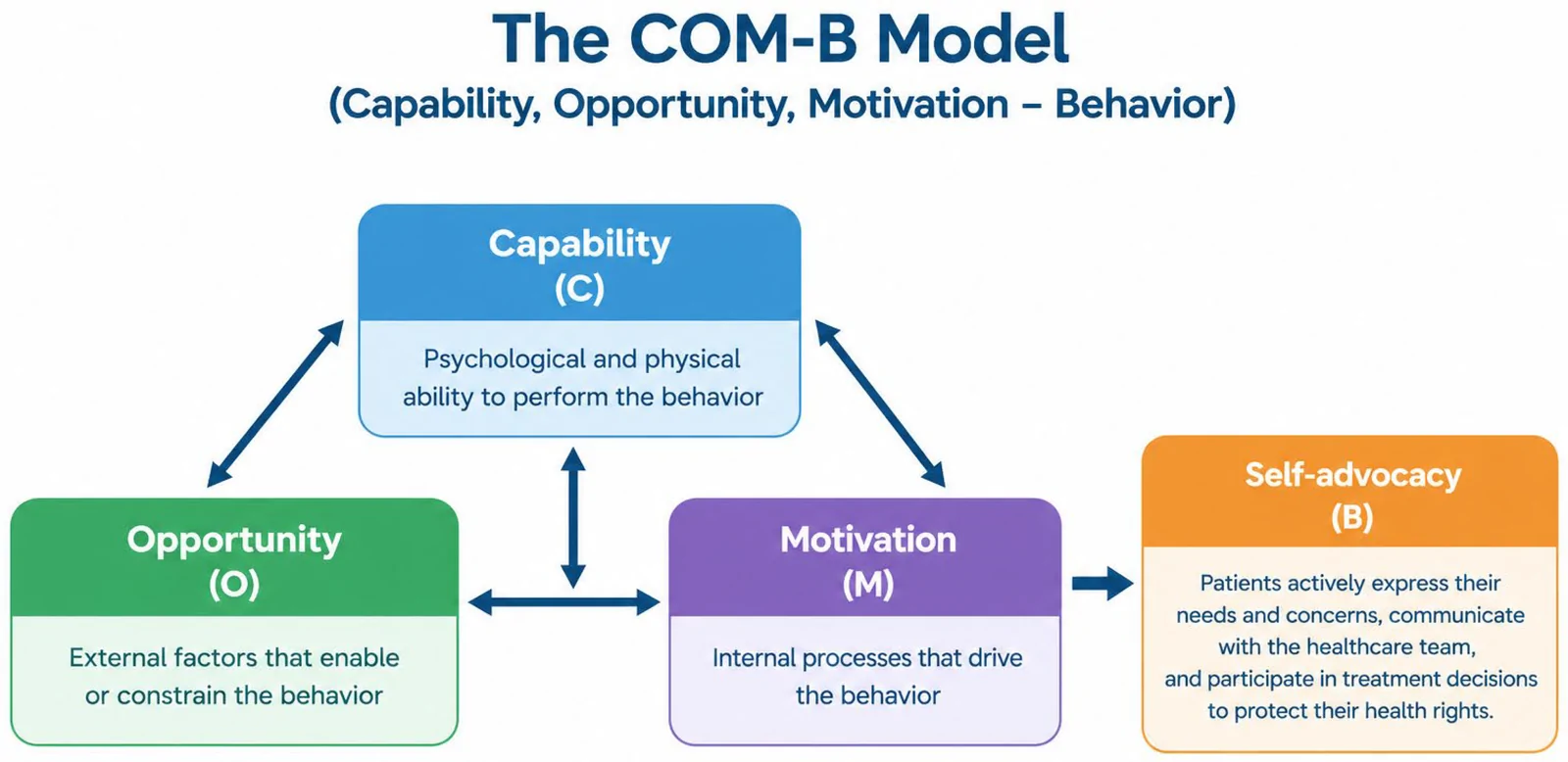
The COM-B Model.

The COM-B model guided both the development of the interview guide and the data analysis process (Figure 1). The interview guide was structured around three dimensions: capability, opportunity, and motivation. The capability dimension focused on patients’ ability to obtain and understand health information, identify their needs, and express their concerns. The opportunity dimension explored external conditions, including family support, the healthcare interaction environment, and opportunities to participate in treatment decision-making. The motivation dimension focused on patients’ perceptions of the value of self-advocacy and psychological factors influencing their behavioral choices. The themes were then mapped and integrated according to the COM-B model to identify capability, opportunity, and motivation factors influencing self-advocacy development among patients receiving MHD. This process used the theoretical framework to deepen understanding of the phenomenon without restricting the emergence of new themes.

### 2.4. Data Collection

Before formal data collection, we invited two patients with MHD who met the inclusion and exclusion criteria to participate in pilot interviews. Based on participant feedback and subsequent team discussions, we added follow-up prompts to selected questions. We did not include pilot interview data in the formal analysis. (see File S2). The first author (JW) conducted all interviews using a semi-structured approach. JW was a postgraduate nursing student with systematic training in qualitative research and clinical experience. JW had no prior relationship with the participants and did not participate in their clinical care. The interviews were conducted in Mandarin, Cantonese, or Teochew, all of which the first author, JW, could speak. Before each interview, JW introduced themself as a postgraduate nursing student and explained the study’s purpose. Participants were told participation was entirely voluntary, they could withdraw at any time, and withdrawal would not affect their subsequent treatment. They were also informed that the interviews would be fully audio-recorded. The researcher obtained written informed consent from all participants before the interviews. The researcher flexibly adapted the interview guide to the circumstances, asking questions in different orders as appropriate. Participants were encouraged to express their thoughts openly, and the researcher documented their nonverbal behaviors during the interviews. Interviews were conducted in a quiet, undisturbed treatment room, with only JW and the participant present.

### 2.5. Data Analysis

Within 24 h after each interview, two researchers (JW and JY) transcribed the audio recordings verbatim and documented participants’ nonverbal behaviors. All participants were invited to review their interview transcripts. Five accepted the invitation, and none suggested any revisions. The researchers transcribed the interview recordings verbatim in Chinese and conducted coding and analysis in Chinese. The researchers managed the transcripts in Microsoft Word 2021 and used Microsoft Excel 2021 to facilitate the coding process. We used an inductive–deductive thematic analysis approach to analyze the data [19]: (1) Inductive phase: Two researchers (JW and JY) repeatedly read the interview transcripts to become familiar with the data and conducted open coding. They generated initial codes directly from the data. The two researchers then compared their interpretations, discussed differences, and deepened their understanding of the data to reach consensus on the initial codes. The team then developed subthemes from these codes. (2) Deductive phase: Guided by the COM-B model, the research team organized and mapped the subthemes generated during the inductive phase to the three dimensions of capability, opportunity, and motivation. These dimensions provided the organizing structure for presenting the findings. The first author (JW) and research team members JY and YZ were all Teochew speakers, while the corresponding author was a Cantonese speaker. During coding, the team discussed and verified expressions with dialectal differences to minimize potential semantic discrepancies from dialect interpretation. After completing the data analysis, the first author translated the interview excerpts selected for the manuscript into English. The corresponding author, who held a second bachelor’s degree in English, checked the translations sentence by sentence against the original Chinese transcripts to identify potential mistranslations, omissions, or semantic discrepancies.

### 2.6. Rigor

Both researchers had received systematic training in qualitative research. Before data collection, the researchers involved in interviewing and coding prepared reflexive statements to document their personal experiences, professional backgrounds, and researcher positions that might influence data collection or interpretation. The interviewer explicitly reported their initial assumptions about the phenomenon under study. These assumptions included that patients receiving maintenance hemodialysis rarely expressed their needs to healthcare professionals proactively and that family members often made decisions on patients’ behalf. During interviews, the researchers used neutral, non-leading prompts to minimize potential influence on participants’ responses. Throughout data analysis, the researchers continually revisited their reflexive notes to consider how their positions and prior assumptions might influence data interpretation and theme development. To further verify the accuracy of the interview data, the researchers returned transcripts to some participants for confirmation. In addition, a researcher not involved in data collection or initial coding reviewed the final coding framework to offer an alternative perspective and identify potentially overlooked aspects of the data.

### 2.7. Ethical Considerations

The Ethics Committee of the First Affiliated Hospital of Guangdong Pharmaceutical University approved this study (Approval No. [2026] IIT No. 15). All participants participated voluntarily and provided written informed consent. Participants were informed that participation was voluntary and that refusal to participate or withdrawal from the study would not affect their subsequent treatment. We de-identified all data, stored them securely, and kept them confidential.

## 3. Results

### 3.1. Demographic and Clinical Characteristics of Participants 

A total of 18 patients receiving MHD (P1–P18) participated in the interviews. Participants were aged 20–73 years, with a dialysis vintage of 5–117 months; 8 were male, and 10 were female. All participants completed face-to-face, semi-structured interviews lasting 25–44 min. No repeat interviews were conducted. Table 1 presents participant characteristics.

### 3.2. COM-B Domains and Themes

Table 2 presents the eight subthemes generated through inductive analysis, together with their corresponding codes and illustrative quotations. Guided by the COM-B model, the eight subthemes were organized under three overarching domains: capability, opportunity, and motivation.

#### 3.2.1. Capability

##### Cognitive Overload

The interviews revealed that when participants received multiple types of information from healthcare professionals, including verbal instructions, treatment plans, medication regimens, and dietary advice, some found it overwhelming and highly technical, making it hard to understand and remember in the moment. Some participants also reported that successive exchanges during clinical encounters, along with other matters requiring their attention, made it difficult to recall the questions they had intended to ask healthcare professionals. These experiences may have hindered their ability to express their questions and needs fully and to participate in related discussions and decision-making.

*“The nurse told me many things to pay attention to during treatment. I thought I understood at the time, but I actually didn’t remember much. I only remembered one or two things.”*

(P4)

*“Sometimes I think about things that happened at home and plan to ask the doctor about them. But once I get to the hospital, there’s the weight and blood pressure checks, the doctor’s rounds, getting my medications, and so on. Then I forget to bring them up.”*

(P9)

*“When the doctor came to see me, he talked about so many things. Before I had even processed the previous sentence, he was already moving on to the next one. My head was buzzing, so I could only nod and say, ‘Okay.”*

(P1)

##### Difficulty Translating Experiences into Expressions

Some patients found it difficult to clearly articulate their bodily experiences, practical needs, or questions, which hindered communication with healthcare professionals.

*“I speak the Teochew dialect, while most of the doctors and nurses here speak Cantonese or Mandarin. I’m not very good at speaking Mandarin, so I usually don’t say much.”*

(P18)

*“I feel uncomfortable, but I don’t know how to describe it. Even now, lying here, my leg feels very uncomfortable, but I don’t know how to put it into words.”*

(P6)

##### Acquisition of Proactive Strategies

During the interviews, some participants described using specific strategies to facilitate communication and verify health information.

*“When I first started dialysis, there were many things I didn’t know. I wrote down the questions I wanted to ask on my phone, so when I saw the doctor, I could show them directly, and she would know what I wanted to ask.”*

(P2)

*“When I don’t understand something, I also ask other patients who have been on dialysis longer. We often chat while waiting for dialysis.”*

(P11)

*“I’m not sure whether the information I learn online is correct, so I write it down and ask my nurse. I only use it after she tells me that it is appropriate for me.”*

(P7)

#### 3.2.2. Opportunity

##### Constrained Patient Voice Within Family Relationships

Some participants reported that during interactions with healthcare professionals, family members spoke directly to them on the patient’s behalf because they were concerned about the patient’s well-being.

*“My mother worries a lot about my health, so she talks to the doctors every time. The doctors have also become used to discussing the treatment plan with her… I’ve gotten used to letting my mother speak for me, so I rarely say anything myself.”*

(P3)

A few patients reported that they tended to express themselves less when their family members did not take their wishes to communicate seriously.

*“My spouse says that I’m old and don’t understand anything, so I should just focus on getting my dialysis. Why ask so many questions? If the doctor has something to say, they’ll tell our son.”*

(P18)

*“I saw a video saying that a certain exercise could improve your health, so I asked my son about it. He said I was always watching people talk about health and then doing things without knowing what I was doing. He told me not to learn these things from videos anymore.”*

(P1)

##### Empowerment Through Continuity of Patient–Provider Relationships

Some participants reported that frequent contact and ongoing interactions with healthcare professionals built mutual familiarity and trust, making them more willing to express their discomfort, questions, or needs directly.

*“Dr. Chen and Nurse Li have been taking care of me all along. We have worked together for several years, and I see them every other day. If I feel uncomfortable, I just tell them directly.”*

(P2)

*“At first, I did not say much. But later, I realized that it was always the same nurse. She remembered which dialysis machine I preferred and always arranged the same bed for me, so I gradually trusted her more. When I had any concerns, I was more willing to tell her.”*

(P6)

*“I have been seeing Director Hu for many years. I first consulted him when I had nephritis, and later he was the one who suggested that I start dialysis. We know each other very well, so I can directly contact him through WeChat if I have any questions.”*

(P8)

#### 3.2.3. Motivation

##### Sensitivity to Relational Costs

Some patients worry that expressing their needs, asking questions, or providing feedback might displease healthcare professionals and, consequently, strain their relationship with them.

*“Sometimes I cannot follow all the instructions the nurse gives me, but I am afraid she will think I am not cooperative and treat me differently later, so I just agree with her.”*

(P17)

*“I feel like having too many questions might annoy them. The doctors and nurses are already very busy, and I do not want to interfere with their work.”*

(P1)

*“I do not really understand these things myself. I am afraid that if I say something wrong, I will embarrass myself, and they might think I do not trust them.”*

(P4)

##### Awareness of Financial Burden

Some patients were worried that expressing their needs might lead to additional medical costs. As a result, they hesitated to voice their concerns or remained silent despite unmet needs.

*“After getting sick, things definitely cannot be the same as before… I feel uncomfortable all over, even my bones hurt. But if I say something, I might need to undergo additional tests. I am not working now, so I just think I should tolerate it if I can.”*

(P10)

*“I used to take lanthanum carbonate for phosphorus control. Later, it was changed to another medication. I felt that the previous medication worked better, but it was also more expensive. I thought that if I could reduce some financial burden for my family, I should do so, so I did not tell the doctor that I wanted to switch back.”*

(P13)

*“I cannot really help my family with anything now. My son is the one supporting the family. How can I still make requests? My child has his own life, and I cannot spend all the family’s money on myself.”*

(P9)

##### Sense of Responsibility for One’s Health

Some patients recognized that maintaining their health required not only support from healthcare professionals but also their own active efforts to monitor their health, acquire relevant knowledge, and speak up promptly when problems arose.

*“My body belongs to me, so I definitely need to pay attention to it. If I do not take the initiative to speak up, how can doctors and nurses take care of every patient when they have so many people to look after? So whenever I feel uncomfortable, I proactively tell them.”*

(P8)

*“After becoming ill, you have to keep learning. If you do not learn, you cannot even understand what the doctor says. If you do not understand your own health condition, how can you know whether these things are suitable for you?”*

(P15)

*“I think dialysis quality also depends on myself. If I find that my phosphorus level is too high, I will pay more attention to my diet and discuss with the doctor whether I should add another hemoperfusion session.”*

(P7)

## 4. Discussion

This study used the COM-B model as a theoretical framework to systematically explore the factors associated with self-advocacy among patients receiving MHD from the perspectives of capability, opportunity, and motivation. The findings describe factors related to health information comprehension, needs expression, family support, patient–provider interactions, and personal motivation, as well as their potential associations with patients’ self-advocacy behaviors. These findings deepen understanding of the complexity of self-advocacy among patients receiving MHD and provide a reference for developing and evaluating relevant support strategies.

This study found that the capability foundation of self-advocacy among patients receiving MHD was mainly reflected in their abilities to process healthcare information, express needs, and apply advocacy strategies. Some participants reported that the volume and technical complexity of the information provided by healthcare professionals made it difficult for them to fully understand and remember it. Cognitive load theory suggests that when the amount of information exceeds the limited capacity of working memory, information processing efficiency decreases, regardless of whether the overload results from inappropriate information presentation or the complexity of the knowledge itself [20]. Patients receiving MHD need to understand not only basic renal pathophysiology but also complex knowledge related to fluid management, dietary restrictions, and vascular access maintenance [21]. Therefore, improving the comprehensibility and reviewability of health information may be an important approach to supporting patients’ involvement in care. For example, tools such as measuring cups for fluid management and physical models illustrating the phosphorus and potassium content of foods [22] could serve as supportive aids and warrant further exploration and evaluation to help patients understand and remember relevant information.

Furthermore, language barriers and difficulties in translating symptoms into words may further disrupt the pathway through which patients communicate meaningful information to healthcare professionals. Consistent language use is a key prerequisite for building patient–provider trust and enabling effective information exchange [23]. Communication training in commonly used local dialects could be offered to healthcare professionals, alongside volunteer interpreters or digital translation tools, to reduce language barriers and help patients communicate their symptoms and needs more accurately. In this study, some patients described using their mobile phones to record questions or seeking advice from peers with longer dialysis experience. These experiences suggest that information aids and peer interactions may help patients understand information, prepare questions, and express their needs. Previous research has also examined the relationship between peer interaction and self-advocacy [24]. Therefore, future research could explore peer activities focused on information exchange and sharing self-advocacy experiences and further evaluate their feasibility and potential effects on patients’ self-advocacy.

This study suggests that both the family environment and the healthcare setting may shape opportunities for self-advocacy among patients receiving MHD. At the family level, some participants described family members communicating with healthcare professionals on their behalf out of concern for their health, or dismissing their desire to express their needs. These experiences may limit patients’ opportunities to express their views directly and participate in care discussions. This finding aligns with Park et al.’s study, which found that excessive family involvement may affect patients’ autonomous decision-making [25]. Under Chinese familism, family members who speak on a patient’s behalf are often viewed as providing care and protection, which can make erosion of patient autonomy less visible in clinical encounters [26]. This support may also reduce patients’ opportunities to participate directly in interactions with healthcare professionals, affecting their self-advocacy. Therefore, care practices should preserve the supportive role of family members while ensuring that patients have opportunities to express their experiences and preferences directly. Family education may also help guide family members to respect patients’ wishes when they choose to express themselves. At the healthcare level, some participants in this study perceived that ongoing contact with relatively familiar healthcare professionals helped build trust and made them more willing to express discomfort and ask questions. Unlike brief encounters in general outpatient settings, patients receiving maintenance hemodialysis typically attend dialysis centers regularly over an extended period. Thomas et al. [27] also noted that continuous patient–provider relationships help maintain continuity in communication and address patients’ communication expectations. These findings suggest that maintaining continuity of care information, attending to previously expressed patient needs, and communicating relevant information appropriately during staff handovers may be worthwhile strategies to explore.

This study found that motivational factors influencing self-advocacy among patients receiving MHD included sensitivity to relational costs, awareness of financial burden, and a sense of responsibility for one’s health. Some patients hesitated to ask questions or share differing opinions because they did not want to challenge healthcare professionals’ authority, aligning with findings from Zhao et al. [28]. In addition, some patients worried that their concerns would interrupt healthcare professionals’ workflow and negatively affect patient–provider relationships, leading them to refrain from expressing their needs. Previous studies in Chinese healthcare settings have also found that patients’ concerns about healthcare professionals’ authority and reluctance to burden physicians may prevent them from expressing their needs and advocating for patient-centred care [29]. In addition, patients experiencing greater financial burdens tend to adopt a more passive stance in healthcare decision-making [30]. As dialysis duration increases, cumulative medical costs can create significant financial stress, leading some patients to suppress care needs to reduce household spending. In contrast, some patients viewed maintaining their health as a personal responsibility and actively sought health information, monitored dialysis-related indicators, and later expressed their needs or suggested treatment adjustments to healthcare professionals. This suggests that a sense of responsibility for one’s health may motivate self-advocacy among patients receiving MHD. Previous research has also emphasized the importance of recognizing the self-empowering actions of people with chronic conditions and creating conditions that facilitate their active involvement in care [31].

Given these factors, future research could explore strategies to promote self-advocacy among patients receiving MHD, focusing on psychological safety, financial support, and empowerment education. First, introduce patient-led agendas during regular ward rounds to enhance patients’ psychological safety through healthcare professionals’ responses and alleviate concerns about potential interpersonal risks. Second, healthcare institutions could strengthen communication about medical insurance and financial assistance policies to help patients understand available resources, such as outpatient reimbursement for chronic diseases and critical illness insurance [32,33]. This may alleviate financial concerns and encourage patients to express their care needs. In addition, health education should help patients gain disease-related knowledge and encourage them to express their care needs proactively. Previous research has reported the potential value of structured self-advocacy education in information seeking, communication, and decision-making participation [34]. Future research could develop self-advocacy education materials tailored to the Chinese dialysis care context and evaluate their acceptability, feasibility, and potential effects.

## 5. Limitations

This study has several limitations. First, participants were recruited from the blood purification centre of a single tertiary hospital. Although maximum variation sampling was used to include participants with diverse demographic and disease-related characteristics, the findings may have limited transferability to other clinical and institutional settings. Second, eligibility required participants to have sufficient language and comprehension abilities to complete the interviews. Consequently, the findings may not fully reflect the self-advocacy experiences of patients with MHD who have substantial communication difficulties, whose experiences also warrant further investigation. Future research could recruit more diverse patient populations across multiple healthcare settings to broaden the understanding of self-advocacy among patients with MHD. Third, the COM-B model guided data collection and analysis. Although this framework facilitated a systematic exploration of factors associated with self-advocacy, its use may have influenced the aspects of participants’ experiences that were explored and interpreted, potentially limiting attention to factors not readily captured by the model. Future research could draw on alternative theoretical perspectives or use more open-ended approaches to explore additional factors associated with self-advocacy and the processes through which they may operate. Finally, this study relied primarily on patient self-reported data. Social desirability bias may have influenced participants’ accounts of sensitive topics, such as patient–provider interactions and the expression of healthcare needs. Although the research team employed multiple strategies to enhance data credibility, some information bias may remain.

## 6. Conclusions

This study used the COM-B model to explore capability, opportunity, and motivation factors associated with self-advocacy among patients receiving MHD. The findings suggest that difficulty understanding health information and expressing personal experiences, along with limited knowledge of self-advocacy strategies, may prevent patients from translating their needs into self-advocacy actions. Excessive family involvement may limit patients’ opportunities to express their preferences, whereas ongoing patient–provider interactions may give them opportunities to voice their concerns. Meanwhile, concerns about interpersonal consequences and financial burden, as well as a sense of responsibility for one’s own health, were also associated with patients’ motivation for self-advocacy. These findings suggest that self-advocacy among patients receiving MHD should be understood not only in terms of individual capability and motivation but also in relation to the opportunities afforded by family relationships, patient–provider interactions, and the broader dialysis care context. Future efforts to support self-advocacy could explore approaches to improve health information communication, strengthen patients’ ability to express their needs, promote supportive family involvement, and improve patient–provider communication. Further evaluation is needed to assess the feasibility of these approaches and their effects on self-advocacy.

## Figures and Tables

**Table 1:** Participant characteristics.

Characteristic	Category	n (%)
Sex	Male	8 (44.4)
	Female	10 (55.6)
Age (years)	20–39	6 (33.3)
	40–59	7 (38.9)
	≥60	5 (27.8)
Education	Primary school	2 (11.1)
	Junior high school	5 (27.8)
	High school/secondary technical school	5 (27.8)
	Junior college/bachelor’s degree	5 (27.8)
	Postgraduate	1 (5.6)
Dialysis vintage (months)	<12	3 (16.7)
	12–24	8 (44.4)
	25–48	5 (27.8)
	>48	2 (11.1)
Marital status	Married	13 (72.2)
	Unmarried	3 (16.7)
	Widowed	2 (11.1)
Employment status	Employed	11 (61.1)
	Unemployed/retired	7 (38.9)
Vascular access	AVF	12 (66.7)
	AVG	2 (11.1)
	CVC	4 (22.2)

Abbreviations: CVC, central venous catheter; AVF, autogenous arteriovenous fistula; AVG, arteriovenous graft.

**Table 2:** Domain and Subthemes with Representative Quotations.

Domain	Subtheme	Code	Illustrative Quotations
Capability	Cognitive overload	Difficulty Retaining Health Information	“The nurses told me many things I needed to pay attention to during treatment. At that time, I seemed to understand, but actually I did not remember much. I only remembered one or two points.” (P4)
	Difficulty translating experiences into expressions	Difficulty Describing Physical Discomfort	“I feel uncomfortable, but I do not know how to describe what I am feeling. For example, when I am lying here, my legs feel very uncomfortable, but I do not know how to explain it.” (P6)
	Acquisition of proactive coping strategies	Proactively Preparing for Communication Using Written Notes	“When I first started dialysis, there were many things I did not know. I wrote down the questions I wanted to ask on my phone, so when I saw the doctor, I could show them directly, and she would understand.” (P2)
Opportunity	Constrained Patient Voice within Family Relationships	Family Members Dismissing Patients’ Perspectives and Communicating on Their Behalf	“My spouse always says that I am old and do not understand anything, and that I should just focus on receiving dialysis. He asks why I need to ask so many questions. The doctor will tell my son if there is anything important.” (P18)
	Empowerment through continuous patient–provider relationships	Continuity of Healthcare Relationships Facilitating Symptom Expression	“It has always been Dr. Chen and Nurse Li (the nurse) who have taken care of me. We have worked together for several years, and I see them every other day. Whenever I feel uncomfortable, I can directly tell them.” (P2)
Motivation	Sensitivity to relational costs	Verbal Compliance Due to Concerns About Negative Healthcare Professional Evaluations	“Sometimes I cannot follow all the advice given by the nurses, but I worry that they will think I am uncooperative and treat me differently in the future, so I just agree with them.” (P17)
	Awareness of financial burden	Refraining from Expressing Needs Due to Concerns About the Family’s Financial Burden	“Now I cannot really help my family with anything. My son is supporting the family, and what right do I have to make more requests? My child also has his own life; we cannot spend all the money on me.” (P9)
	Sense of Responsibility for One’s Health	Taking Responsibility for One’s Own Health and Proactively Reporting Discomfort	“My body belongs to me, so I have to take responsibility for it. If I do not speak up, the doctors and nurses have so many patients to take care of, how could they notice everything? Therefore, whenever I feel uncomfortable, I actively tell them.” (P8)

## Data Availability

The qualitative data supporting the findings of this study may be available from the corresponding author upon reasonable request, subject to ethical approval and participant confidentiality requirements.

## Supplementary Materials

The following supporting information can be downloaded at: https://www.mdpi.com/article/10.3390/healthcare14213758/s1, File S1: Consolidated criteria for Reporting Qualitative research Checklist; File S2: Interview Guide: “Exploring Factors Related to Self-Advocacy Among Patients Receiving Maintenance Hemodialysis in China: A Qualitative Study Based on the COM-B Model”.

## Abbreviations

The following abbreviations are used in this manuscript:

MHD	maintenance hemodialysis
COM-B	capability, opportunity, motivation–behavior
CVC	central venous catheter
AVF	autogenous arteriovenous fistula
AVG	arteriovenous graft
