# Supplementary material for: Exploring Factors Related to Self-Advocacy Among Patients Receiving Maintenance Hemodialysis in China: A Qualitative Study Based on the COM-B Model
Source: Healthcare (Basel). 2026 Nov 4;14(21):3758. doi: 10.3390/healthcare14213758 (PMC13763094; doi:10.3390/healthcare14213758)
Supplement: Supplementary file 1 [file healthcare-14-03758-s001.zip › File S2.pdf]

Interview Guide: "Exploring Factors Related to Self-Advocacy Among Patients Receiving Maintenance Hemodialysis in China: A Qualitative Study Based on the COM-B Model"

Interview Guide	
Capability	During dialysis, if you have questions about your treatment, physical condition, or nursing care, how do you usually seek information or address them? Do you actively ask doctors or nurses?
	When you experience physical discomfort during treatment or have your own thoughts about your treatment or nursing care, how do you usually express them to doctors or nurses? What difficulties do you encounter when expressing your thoughts?
Opportunity	During dialysis treatment, what people or environmental factors influence your ability to express your thoughts and needs?
	When your treatment plan or nursing care is being discussed or arranged (e.g., medication adjustments, dialysis-related arrangements, or symptom management), how do doctors and nurses usually communicate with you?
Motivation	What impact do you think expressing your needs or thoughts to doctors and nurses may have? Does this affect your willingness to express them in the future?
	When your thoughts or needs differ from those of doctors or nurses, what factors do you usually consider?
